# Splitting or Lumping? A Conservation Dilemma Exemplified by the Critically Endangered Dama Gazelle (*Nanger dama*)

**DOI:** 10.1371/journal.pone.0098693

**Published:** 2014-06-23

**Authors:** Helen Senn, Lisa Banfield, Tim Wacher, John Newby, Thomas Rabeil, Jennifer Kaden, Andrew C. Kitchener, Teresa Abaigar, Teresa Luísa Silva, Mike Maunder, Rob Ogden

**Affiliations:** 1 WildGenes Laboratory, Royal Zoological Society of Scotland, Edinburgh, United Kingdom; 2 Conservation Department, Al Ain Zoo, Al Ain, Abu Dhabi, United Arab Emirates; 3 Conservation Programmes, Zoologicial Society of London, Regents Park, London, United Kingdom; 4 Sahara Conservation Fund, L'Isle, Switzerland; 5 Sahara Conservation Fund, Niamey, Niger; 6 Department of Natural Sciences, National Museums Scotland, Chambers Street, Edinburgh, United Kingdom; 7 Institute of Geography, School of Geosciences, University of Edinburgh, Drummond Street, Edinburgh, United Kingdom; 8 CIBIO/InBIO, Centro de Investigção em Biodiversidade e Recursos Genéticos da Universidade do Porto, Vairão, Portugal; 9 Estación Experimental de Zonas Áridas, Consejo Superior de Investigaciones Científicas (CSIC), Almería, Spain; 10 Departamento de Biologia da, Faculdade de Ciências da Universidade do Porto, Porto, Portugal; 11 College of Arts and Sciences, Florida International University, Miami, Florida, United States of America; State Natural History Museum, Germany

## Abstract

Managers of threatened species often face the dilemma of whether to keep populations separate to conserve local adaptations and minimize the risk of outbreeding, or whether to manage populations jointly to reduce loss of genetic diversity and minimise inbreeding. In this study we examine genetic relatedness and diversity in three of the five last remaining wild populations of dama gazelle and a number of captive populations, using mtDNA control region and cytochrome b data. Despite the sampled populations belonging to the three putative subspecies, which are delineated according to phenotypes and geographical location, we find limited evidence for phylogeographical structure within the data and no genetic support for the putative subspecies. In the light of these data we discuss the relevance of inbreeding depression, outbreeding depression, adaptive variation, genetic drift, and phenotypic variation to the conservation of the dama gazelle and make some recommendations for its future conservation management. The genetic data suggest that the best conservation approach is to view the dama gazelle as a single species without subspecific divisions.

## Introduction

Fragmented populations of endangered species present a conservation dilemma [1]. If numbers dwindle to the extent that conservation intervention is necessary, should they be managed separately to conserve locally adapted genetic diversity and minimise the risk of outbreeding? Or should they be managed jointly to preserve evolutionary potential and to reduce the risk of inbreeding? This dilemma has been discussed recently both from taxonomic [2]–[3] and management [4] perspectives and is present in many wild and captive management scenarios [5]–[10]. The dilemma is typified in regard to the Critically Endangered dama gazelle (*Nanger dama*) [11].

**Figure 1:**
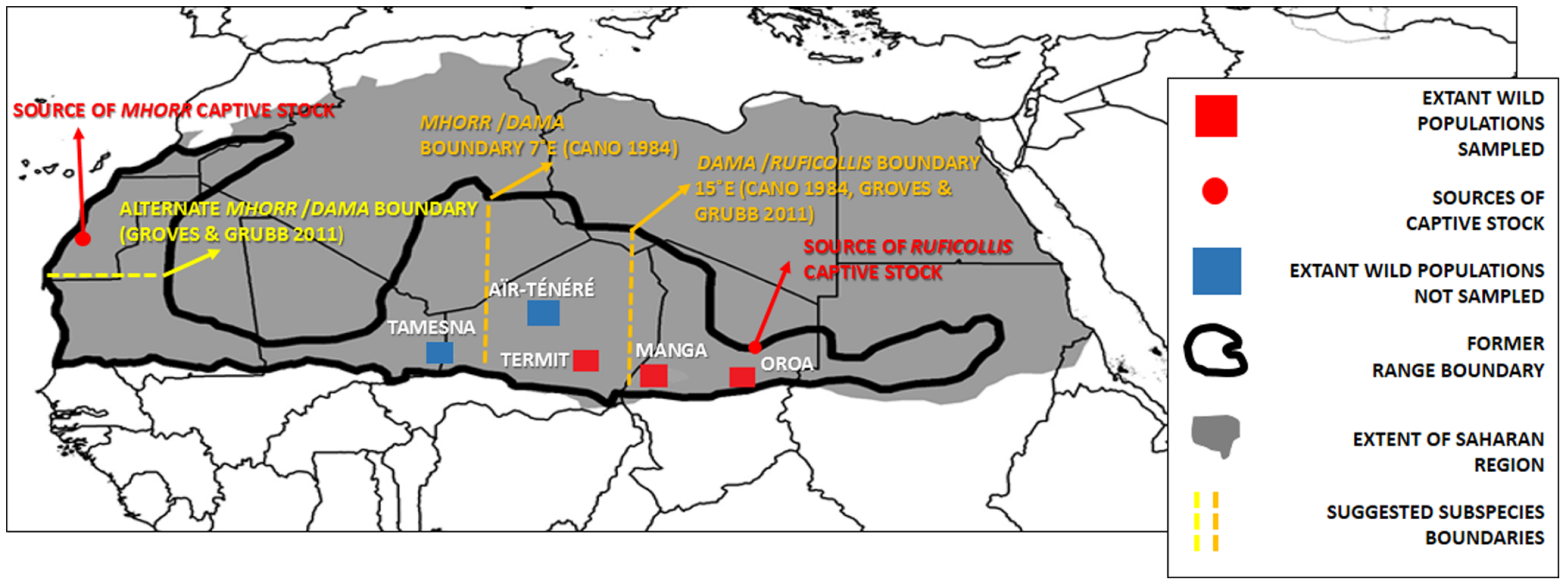
A map depicting the former and current range of the dama gazelle (adapted from [14]). Wild sampling sites and places of origin for the captive populations are depicted. The suggested subspecies boundaries according to different authors are listed (see in text for detail).

There are around 300 dama gazelles left in the wild [11]–[12] and their range has shrunk by 99% during the period 1960–90 [13]–[14]. They now only exist in confirmed isolated populations in Chad, Niger, and Mali (Figure 1) and possibly in Sudan. Approximately 550 individuals are thought to exist within zoos and breeding centres across Europe, the USA and the Middle East, with further populations in private collections in the Middle East and particularly the USA accounting for an estimated 1000 individuals.

The gazelle's range formerly spanned the entire Sahel and Atlantic Saharan region (Figure 1) from south of the Anti-Atlas Mountains to the west coast of Morocco, south to Senegal and Mauritania, and east to the River Nile in Sudan. It is likely this range was once continuous (Figure 1), although a possible break in southern Mauritania has been debated [15]–[16]. The current wild populations are situated in the central part of the former range. Captive populations are known to have originated from two distant points of the original geographical distribution and are currently maintained as separate breeding populations (Figure 1, Table 1).

**Table 1:** Details of the 124 samples in this study and the populations that they originated from.

Population	Site Code (suffix indicates putative subspecies)	Details	Putative sub-species	Number of Samples collected
WILD				
Chad (Ouadi Rimé-Ouadi Achim)	OROA_R	Wild Population in Ouadi Rimé-Ouadi Achim Game Reserve in Central Chad(∼N14.9027, E19.8318)	*N.d.ruficollis*	18
Chad (Manga)	MANGA_R	Wild Population in Manga region of Western Chad (∼N15.33087, E15.1277)	*N.d.ruficollis*	6
Niger (Termit)	TERMIT_D	Wild population(s) in the Central (∼N16.1047, E11.4171) & Northern (∼N16.3706, E11.4581) massif of the Termit mountains	*N.d.dama*	12
ZOO/CAPTIVE				
Al Ain Zoo ‘*mhorr*’	AIN_M	Origin unrecorded, highly likely to be descended from animals in the EEP (originally from EEZA).	*N.d.mhorr*	42
Al Ain Zoo ‘*ruficollis*’	AIN_R	Origin unrecorded, likely to stem from the North American Regional Studbook for addra (*ruficollis*) gazelle as it records the transfer of two females and a male to Al Ain Zoo in 1982.	*N.d.ruficollis*	20
Dama gazelle EEP	EEP_M	Animals sampled from City of Belfast Zoo, EEZA and Montpellier all ultimately originating from EEZA	*N.d.mhorr*	12
Marwell Zoo ‘*ruficollis*’	MAR_R	Origin is the North American Regional Studbook for addra (*ruficollis*) gazelle.	*N.d.ruficollis*	5
Katané, Ferlo North Game Reserve, Senegal	SEN_M	Ultimately originating from EEZA via Réserve Spéciale de Faune de Guembeul, Senegal	*N.d.mhorr*	3
Safia Reserve, Morocco	SAF_M	Ultimately originating from EEZA via R'Mila Royal Reserve, Morocco.	*N.d.mhorr*	6

**Figure 2:**
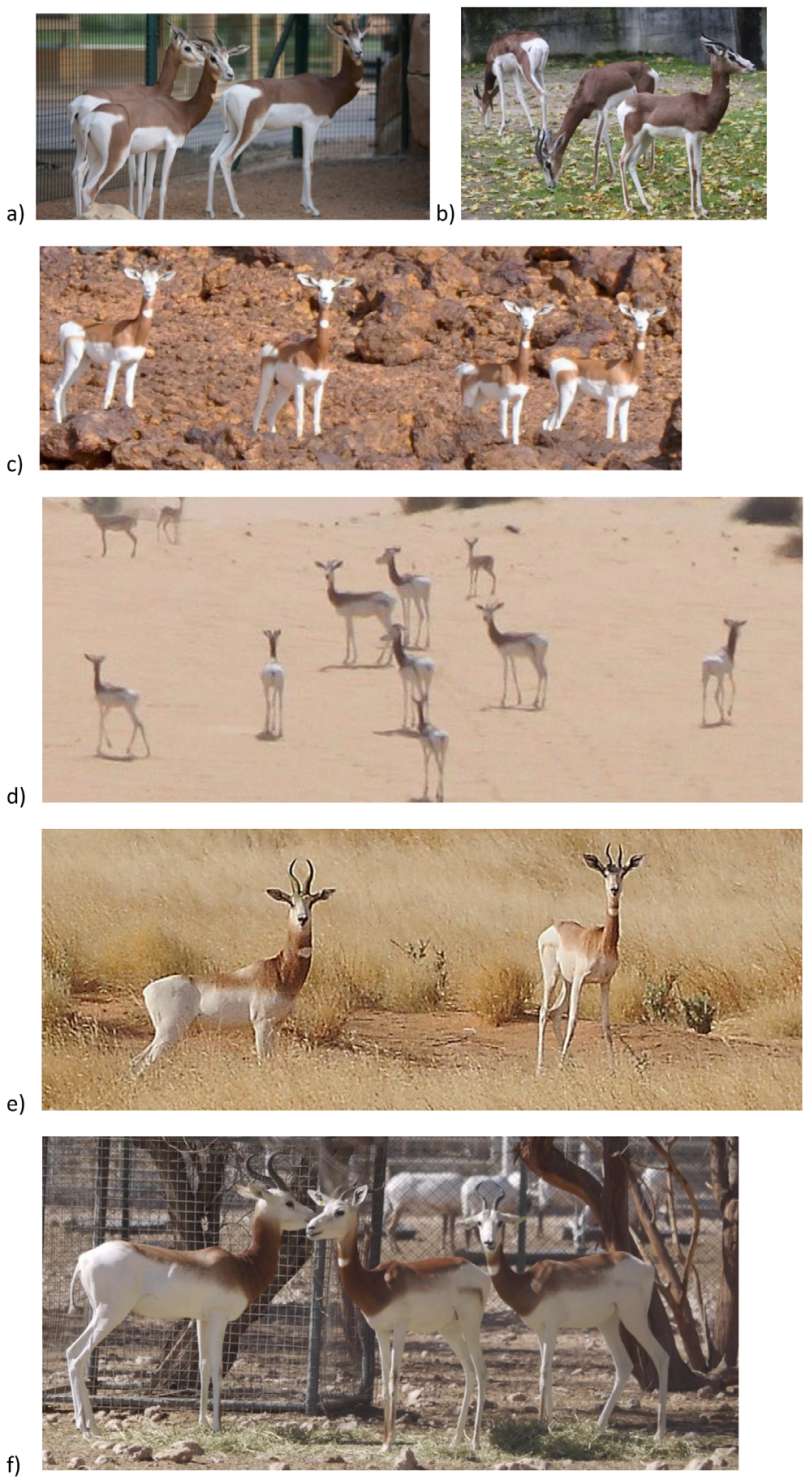
Variation in pelage coloration in different populations listed from northwest to southeast. a&b) Captive *mhorr* at Al Ain (a) and Frankfurt Zoo (b) which are descended from four founders caught in the Dora-Hagunia and Tichla-Bir Ganduz area of Western Sahara in 1958 (in the case of Al Ain zoo the origin is unrecorded, but it is highly likely that this is where they come from). c) animals from the population in Termit, Niger. d) animals from the population in Manga, Chad. e) animals from the population in Ouadi Rimé-Ouadi Achim, Chad. f) animals from the captive population at Al Ain Zoo, most likely descended from 20 founders taken from the wild in around Ouadi Haouach close to Ouadi Rimé-Ouadi Achim, Chad. Animals from the most north-westerly populations have the most extensive dark coloration, which descends down the legs. Moving to the south and east, this dark coloration fades upwards and forwards. Note that there is also phenotypic variation within populations, for example the width of the thigh marking differs in Manga and the animals in OROA exhibit presence or absence of the ham-shaped mark on the thigh [15].

The dama gazelle is the largest of the gazelle species [17]. It shares the genus *Nanger* with Soemmering's gazelle (*N. soemmerringii)* and the Grant's gazelle complex (*N. granti* and related species) [18]. Once previously included in the genus *Gazella*, molecular phylogenetic analysis revealed inclusion of these larger gazelles would make the genus *Gazella* paraphyletic and hence they have been separated into the genus *Nanger* [19]. The dama gazelle has been subdivided into a variable number of subspecies (see below). The coloration of the dama gazelle's pelage is variable and, according to taxonomic tradition, was described by Cano [15] as following a northwest to south and east cline across the species' range: Animals in the east are predominantly white with reddish-brown coloration confined to the neck and shoulders, whereas animals in the west have extensive reddish-brown coloration, which extends further down the back, flanks and haunches. In the far north-west the animals are predominantly chestnut with a white underbelly and rump (Figure 2). However, this is an over-simplification of the dama gazelle's geographical variation, especially in the center of its range. Among former and remaining wild populations in Chad, considerable individual variation in the extent of the dark dorsal coloration was and is obvious even within the same herd [20]–[21].

### Geographical variation and putative subspecies

This apparent cline in dorsal coloration led to the designation of a number of subspecies, based mostly on single specimens, which are often poorly described [22]–[25]. Cano [15] examined 50 museum specimens and captive populations to determine only three subspecies, a classification that is followed generally today: *N. dama ruficollis* occurs east of c.15°E, *N. dama dama* occurs from c.7°E to c.15°E, and *N. dama mhorr* occurs west of c.7°E. This tri-part classification was also followed by Groves and Grubb [18], although they presented a difference in the location of the boundaries between *dama* and *mhorr* locating it further northwest, stating that *mhorr* did not occur further south than the Western Sahara (Figure 1). This difference is due to historical confusion over the type locality of the species, then “*Antilope dama*” described by Pallas in 1766 [22] based on a specimen described and figured by Buffon in 1764 [23] which was collected from Senegal. It was later assumed that this specimen had in fact been collected in the Lake Chad region and not Senegal based on the pelage coloration [15]. Groves & Grubb [18] argue that it is more likely however that it did indeed come from Senegal because Lake Chad was not discovered by Europeans until 1823, more than 50 years after the species was described. The figure by Buffon [23] is, however, not a very biologically accurate representation of the species, illustrating the difficulty of making reliable inferences from historical references. Owing to the confusing number of subspecies names described for this species, we reproduce here the synonyms that Groves and Grubb [18] provide for each putative subspecies:

*N. d. dama* (Pallas, 1766), including *Antilope nanguer*, *Antilope dama* var. *occidentalis*, *Gazella dama permista*, *Gazella mhorr reducta*, *Gazella dama damergouensis*, *Gazella dama weidholtzi*

*N. d. ruficollis* (Hamilton Smith, 1827), including *Antilope addra*, *Antilope dama* var. *orientalis*

*N. d. mhorr* (Bennett, 1833), including *Gazella dama lozanoi*

*N. d. mhorr* is often referred to by the common names of mohor or mhorr gazelle and *N.d. ruficollis* is often called the addra gazelle. Here we use the term “dama gazelle” to cover all putative subspecies and populations of the species *Nanger dama* and for the time being we follow Cano's [15] definition of subspecies and Groves and Grubb's [18] geographical ranges for each subspecies. We highlight here again however that considerable variation within the subspecies has been recoded recently [12] [21] and that [20] observed phenotypic variation, claiming to have seen representatives of all three subspecies phenotypes within Eastern Chad.

### History of captive populations

The early history of the captive stock is detailed by Cano [26]. In 1971, a captive breeding programme was established for *N. d. mhorr* in Almeria (Spain) at the Parque de Rescate de Fauna Sahariana (now known as “La Hoya” Experimental Field Station, Estación Experimental de Zonas Aridas), with animals that had originated from the Dora-Hagunia and Tichla-Bir Ganduz of Western Sahara in 1958 [26]–[27]. The last remaining *N. d. mhorr* were seen there in 1968 [26]. The captive population was based on one male and three females, which are the founders for all animals within the mhorr gazelle international studbook. There are currently 293 animals in 20 institutions across Europe, USA, Africa and the Middle East, of which the largest collection, ∼100 individuals, is at the original location of the “La Hoya” Experimental Field Station. Additionally, this captive population has been either the direct or indirect source (via descendants in other institutions) of all animals for a number of reintroductions to fenced protected areas in North and West Africa; Bou Hedma National Park, Tunisia (22 animals in the early 1990s) [28]–[29]; Souss-Massa National Park, Morocco (12 animals in 2006); Domaine Royal de R'Mila, Morocco, Safia Reserve, Morocco; Guembeul Faunal Reserve, Senegal (seven gazelles in 1984) [30], of which five descendants were the founders for the population at Katané, Ferlo North Game Reserve, Senegal, in 2003 [31]. There are no other known sources of mhorr gazelles, so that in all likelihood the entire captive population today is descended from just four founders.

The decision was taken in 1995 by the European Association of Zoos and Aquaria (EAZA) (whose animals had originally come from USA populations) to phase out the captive management of *N. d. ruficollis* “addra” in favour of *N. d. mhorr* [32]. Similarly, AZA took the decision in 2008 to phase out management of *N. d. mhorr in* favour of *N. d. ruficollis*. Therefore, the current situation is that *N.d.ruficollis* is subject to coordinated breeding efforts in the USA and breeding of *N.d.mhorr* is coordinated in Europe. A number of institutions in the Middle East hold both subspecies

There has been historical debate over whether the *ruficollis* population is indeed *ruficollis* or in fact *dama* or a mixed subspecies population, but here we refer to it as *ruficollis* throughout, following the current consensus. Twenty-eight institutions across the USA hold a total of 168 animals [33]. The studbook was founded by five females and three males from the Ouadi Haouach region of north-east Chad [34], which crosses the north-east boundary of the Ouadi Rimé-Ouadi Achim Game Reserve sample site in this study (see OROA Table 1). Therefore, this captive population (which shows variation in coat coloration) is defined as *ruficollis*, because the original collecting locality is within this subspecies' range according to the Cano [15] classification and comes from a location close to where an extant wild dama population shows marked inter-individual variation in detail of basic pelage pattern. There are also a similar number of *N. d. ruficollis* in captivity in private collections, for which no additional founders are known.

Given the small number of animals left in the wild, the studbook-managed populations represent a considerable proportion of the species' global population (∼300 in the wild, ∼550 in captivity). At least as many animals again exist in captivity outside the two studbooks, notably on private ranches in Texas [35]. So if there can be any positive news for this species at all, it is the captive insurance populations are reasonably large in size. However, it cannot be assumed that all animals alive are currently of equal genetic value to the long-term conservation of this species. Both wild and captive populations are likely to be experiencing higher rates of inbreeding relative to the past (wild populations due to reduction in numbers and fragmented populations, and captive populations due to the small number of founders). However, levels of inbreeding (and inbreeding depression) may differ considerably between populations. Additionally remaining (wild and captive) populations are unlikely to be equally closely related to each other.

### Study Aims

The purpose of this study was to assess the relatedness and relative genetic diversity in extant populations and to use this information to assess the conservation value of different populations and the validity of the current subspecies classification system in order to inform future conservation management strategies for the dama gazelle.

## Methods

### Sample collection

In total 124 samples were collected. This included 36 samples from the wild that were collected as a result of six collecting trips on public land between 2009 and 2013 with the authority and participation of the Termit & Tin Toumma National Nature Reserve, Niger and the Ouadi Rimé-Ouadi Achim Game Reserve and Government of Chad. Samples were collected opportunistically as pellets from distinctly separated fresh piles of faeces, in most cases following direct observation of wild dama gazelles. Samples from captive and reintroduced populations represent a mixture of blood and tissue samples taken from routine veterinary treatment or during autopsy (following natural death) or via faecal sampling and were provided by the relevant institutions or from the collections of National Museums Scotland (see Supplementary Table S1 for details of specific samples), and as a result of two collection trips to Safia and Katané reserves (public land) with the permission of the National Parks in Senegal and the Haut Commissariat des Eaux et Fôrets et de la Lutte Contre la Desertification in Morocco (Table 1: summary details of samples used in this study). CITES permits were in place for the samples that required them. No animals were harmed or killed for the purpose of this study.

### DNA extraction

Tissue and blood samples were extracted using DNeasy blood and tissue kits (Qiagen) and faecal samples with the QIAamp DNA Stool Mini Kit (Qiagen), according to standard protocols. In the case of faecal samples, a single pellet was used per extraction.

### Control region mtDNA sequencing

Primers were designed from existing sequences on Genbank to amplify a 560 basepair fragment from the centre of the control region: nang-fwd (5′ to 3′): cta tgt cct gtt acc att gac , nang-rev (5′ to 3′): gat tgt cca cat gca tat aag c . PCR amplification of the fragment was conducted with 1 µl of template DNA (10–50 ng ml^−1^)1 µl each of forward and reverse primer (10 µM) and 7 µl of Maxima Hot Start PCR Master Mix (Thermo-Fisher). Amplification was performed with an initial denaturation step of 5 mins. at 95°C, followed by 35 cycles of 1 min. (denaturation) at 95°C, 1 min. (primer annealing) at 58°C, 1 min. (elongation) at 72°C and ending with a 72°C extension for 10 mins. Negative controls were run as standard.

The fragments were examined by running them out on a 1% agarose gel and successfully amplified products were cleaned up by addition of 0.5 µl of the enzymes EXO1 and FastAP (Fisher) with an incubation step of 37°C×45 min and a denaturation step of 80°C×10 min. Fragments were sequenced in the forward direction using the BigDye Terminator Kit® (Applied Biosystems), using 3 µl of PCR product and conditions according to manufacturer's instructions. Sequences were run on a capillary ABI 3730 DNA Analyzer sequencer® (Applied Biosystems). In the case of faecal samples, sequencing was additionally conducted in the reverse direction and for a subset of samples with unique haplotypes the PCR was repeated and sequenced.

### Cytochrome b sequencing

Since cytochrome b evolves at a slower rate than the control region, a subset of 16 samples exhibiting unique control region DNA haplotypes were sequenced for a 421 base pair region of the mtDNA cytochrome b gene using the primers MCB 398 and MCB 869 [36]. PCR reaction volumes were identical to those for the control region: thermocycling conditions were: were 95°C×5 min./[95°C×30 s, 50°C×90 s, 72°C×30 s]×35 cycles/60°C×30 min. PCR clean-up and sequencing were conducted as for the control region (see above). Sequencing was conducted in a single direction, using the primer MCB 398 and verified in the reverse direction for a subset of unique haplotypes.

### Alignment

Chromatograms were analysed with Geneious Pro v6.1.4 [37]. Primer sequences were trimmed from the alignments. The cytochrome b sequences were translated to verify the absence of stop codons in the sequences. Sequences aligned with the ClustalW algorithm implemented in the Geneious Pro software and final corrections done by eye.

### Tree and network building

Tree building was performed separately for control region and cytochrome b fragments, using two different methods for each fragment. Firstly, a simple distance-matrix method was used to build the trees. This was done using neighbour joining with the Tamura-Nei model of genetic distance. The trees were built in Geneious Pro v6.1.4 and one-hundred bootstrap iterations were performed. Secondly, Bayesian inference was carried out with MrBayes v3.1.2 [38]. Posterior probabilities were calculated using four heated Markov chains run (chain temp. 0.2) for 2,000,000 Metropolis-coupled MCMC generations. Tree sampling was conducted every 500 generations and a burn-in of 200,000 trees was used. The model used for nucleotide substitution was the General Time Reversible model with among-site substitution-rate heterogeneity described by a gamma distribution and a fraction of sites constrained to be invariable (GTR+Γ+I). This was chosen as a conservative measure, because it has been previously been shown that Bayesian inference of phylogenetic trees performs better, if the chosen substitution model tends toward being over- rather than under- parameterised [39]. The Bayesian analysis was replicated twice to examine its stability.

Control region trees were built using the Genbank haplotype JN632666 (*Nanger granti*) as an outgroup and incorporating sequence JN632665 from a dama gazelle, originating from San Diego Zoo. Here it is referred to as control region haplotype S.

Haplotype networks of the control region data were created using TCS v1.21, treating gaps as a 5^th^ state (there is in fact only a single in-del in the alignment). The network was examined at a number of different probabilities of parsimony (Templeton *et al.* 1992).

Cytochrome b trees were built, including Genbank sequences KC188777, JN632667, JF728776 (*Nanger soemmerringii*) and JN632666 (*Nanger granti*) as outgroups. Sequence JN632665 from the same dama gazelle, originating from San Diego Zoo, was also incorporated into the tree [40]. Here it is referred to as cytochrome b haplotype 4.

### Nucleotide diversity

Haplotype and nucleotide diversity and matrices of mutational distance between haplotypes were calculated using Arlequin 3 [41].

## Results

### Control region

**Figure 3:**
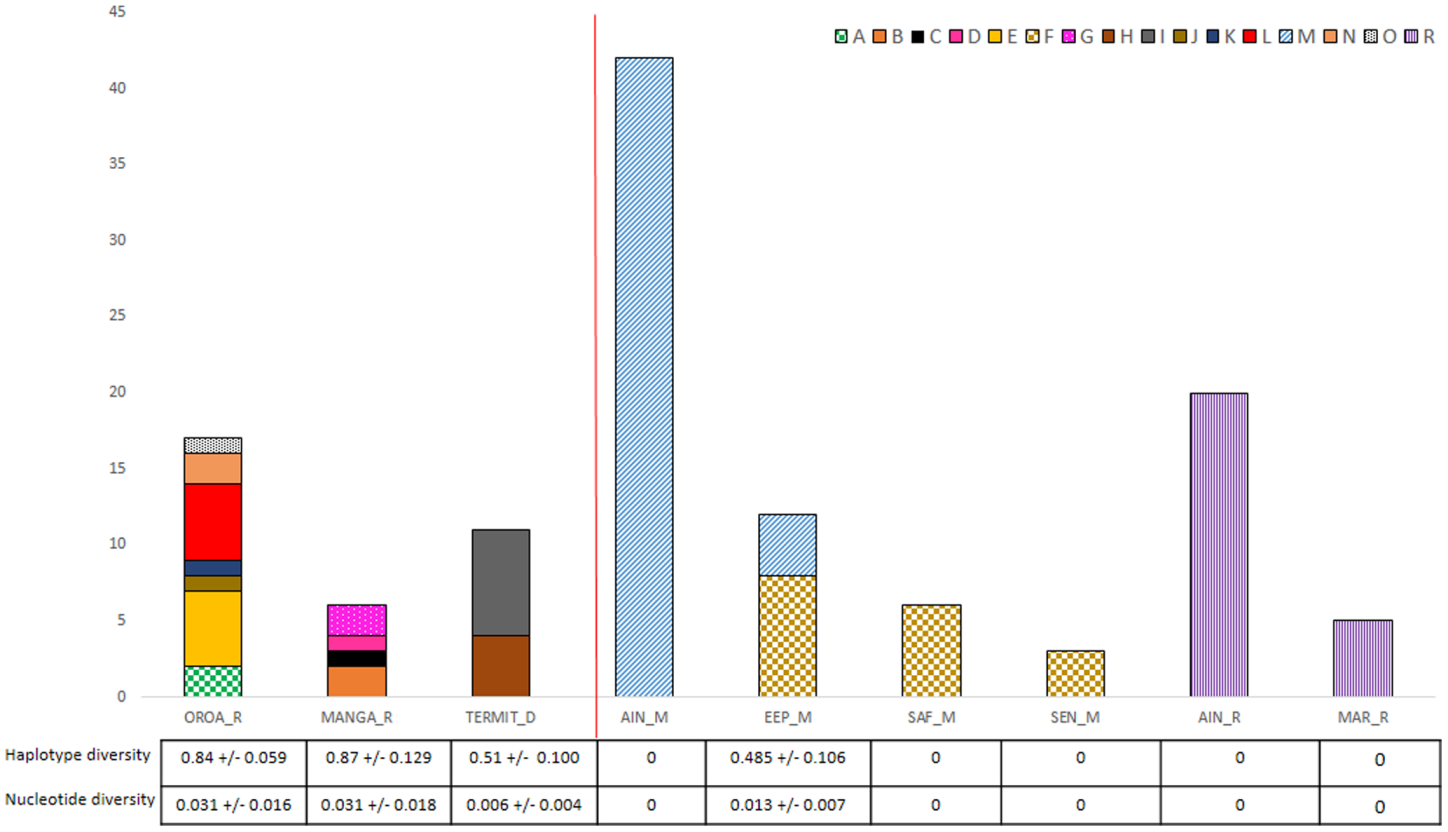
Bar chart of control region haplotypes (A–O & R) found at different sampling sites in this study. Sites are enumerated by collecting locality (see Table 1) and putative subspecies; R (*N. d. ruficollis)*, D (*N. d.* dama), M (*N. d. mhorr*). Wild and captive populations are separated by a red line. Particularly striking, but not unexpected, is the higher haplotype diversity in the samples from wild populations (OROA_R, MANGA_R,TERMIT_D) than in samples from captive and captive-derived *mhorr* populations (Ain_M, EEP_M, SEN_M, SAF_M) and captive *ruficollis* populations (Ain_R, Mar_R). No haplotypes are shared between wild populations, or between wild and captive populations.

In total, 122 control region sequences were obtained from the 124 samples, with only two samples failing to give any result (they were faecal samples collected from the sites EEP_M and OROA). 16 haplotypes were discovered and these sequences have been named A to O & R and have been placed on Genbank under accession KJ848615–30. The majority of the haplotype diversity was spread between the wild populations (13 haplotypes). Captive and captive-derived populations (SEN_M, SAF_M) only contained three haplotypes, two within *mhorr* and one within *ruficollis*. No haplotypes were shared between the different wild populations, or between wild and captive populations (Figure 3). Haplotype diversity [42], defined as the probability that two randomly chosen haplotypes are different in the sample, was highest in the populations OROA and Manga (0.840+/−0.059 s.d., 0.870+/−0.129 s.d.) and lower, but roughly equivalent between the Niger and EEP_M populations (0.510+/−0.100 s.d., 0.485+/−0.106 s.d.) (Figure 3). Nucleotide diversity, defined as the probability that two randomly chosen homologous nucleotides are different, was highest in OROA and Manga (0.031+/−0.016 s.d., 0.031+/−0.018 s.d.). Nucleotide diversity was somewhat higher in the EEP_M (0.013+/−0.007 s.d.) than in Niger (0.006+/−0.004 s.d.) (Figure 3).

For the Bayesian inference of phylogeny the average standard deviation of split frequencies was 0.0069 after 2,000,000 Metropolis-coupled MCMC generations for both of the two replicates.

**Figure 4:**
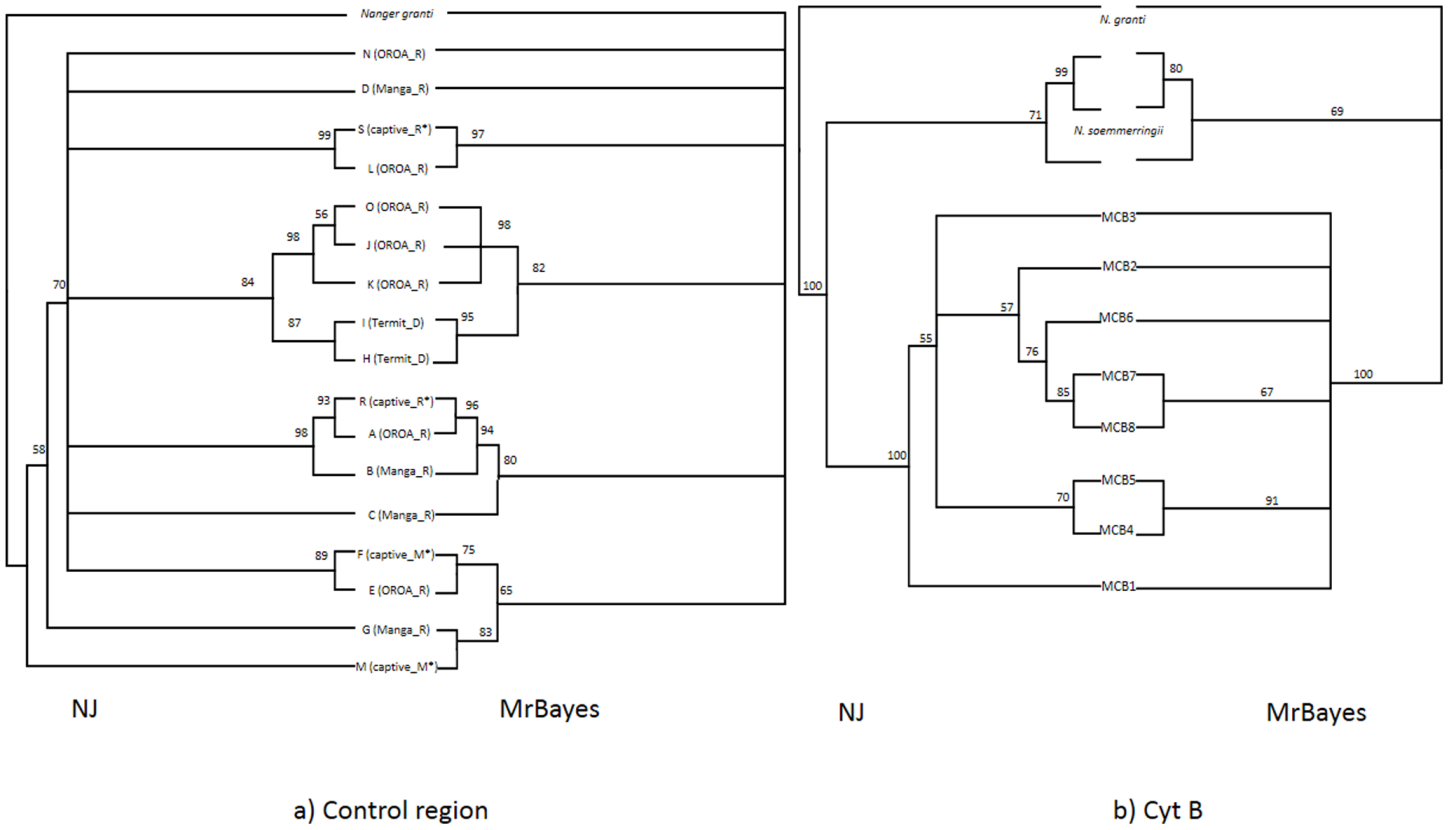
Evolutionary relationships at different genes a) Tree based on 560 bp of control region and b) 421 bp of cytochrome B. In each case Neighbourhood joining (left) and Bayesian Inference of Phylogeny via MrBayes (right) were conducted according to the conditions listed in the methods section. The putative *ruficollis* subspecies exhibits polyphyly at the control region (and cytochrome b, compare with Figure 5). * Captive populations have been combined (see Table 1).

Phylogenetic analysis of the mtDNA control region data revealed tree topologies that were roughly concordant with Neighbour-Joining and Bayesian methods. However, support was generally low apart from for clusters (S, L), (O,J,K), (H,I) and (R, A,B) (Figure 4a).

**Figure 5:**
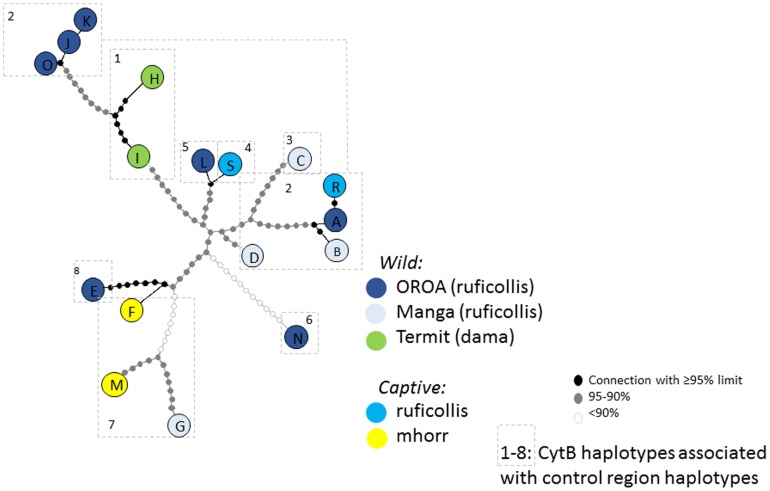
Haplotype network of the control region haplotypes present in this study. Each haplotype is colour coded according to population of origin, and single base-pair step-wise mutations between haplotypes are colour coded according to their connection limit. Relatedness of haplotypes does not correspond to subspecies divisions or geographical structure.

Network analysis revealed a similar certainty surrounding resolution, with four clusters being resolved at a 95% connectivity limit (S,L), (H,I,K,J,O), (R,A,B) and (E,F). Relaxation of the connectivity criteria to below 90% was required to draw a complete network, with 18 mutational steps required to link the entire tree (Figure 5). Pair-wise differences between control region haplotypes can be found in Table 2.

**Table 2:** Matrix of pair-wise mutational differences between control region haplotypes.

	A	E	K	J	O	L	N	C	B	D	G	I	H	M	F
**A**															
**E**	23														
**K**	17	20													
**J**	18	21	1												
**O**	20	21	3	2											
**L**	19	22	18	19	19										
**N**	22	25	19	20	22	21									
**C**	16	20	19	20	22	19	21								
**B**	3	22	18	19	21	18	21	15							
**D**	14	17	15	16	18	11	18	14	15						
**G**	26	21	21	22	22	17	24	25	23	22					
**I**	22	23	15	14	14	15	22	18	21	14	22				
**H**	25	22	14	13	13	22	23	23	22	21	23	7			
**M**	26	21	25	26	26	25	28	29	25	24	12	26	25		
**F**	19	8	18	19	19	16	19	20	18	13	17	19	20	15	
**R**	2	23	19	20	22	21	22	17	5	16	26	24	27	26	19

### Cytochrome b

In total seven haplotypes of cytochrome b were discovered, which were named MCB1–3, 5–8 and placed on Genbank under accession KJ848631–7. Individuals carrying control region haplotypes H and I corresponded to MCB Haplotype 1. MCB2 corresponded to control region B,O,A,D,K,J and R; MCB3 to C; MCB5 to L; MCB6 to N, MCB7 to G,M,F and MCB 8 to E. As previously mentioned, MCB4 corresponds to S (JN632665 whole mitochondrial DNA sequence from animals from San Diego Zoo). These cytochrome b haplotype groupings have been overlaid on the control region network (Figure 5).

For the Bayesian inference of phylogeny the average standard deviation of split frequencies was 0.007 and 0.0042 after 2,000,000 Metropolis-coupled MCMC generations for the two replicates. Graphical output of the change in Log Likelihood during the analysis can be found in supplementary material 1.

Resolution of the phylogeny was not much improved at the cytochrome b gene (Figure 4b), with only a clustering of haplotypes 4 and 5 showing strong support (Figure 4b).

Pair-wise differences between cytochrome b haplotypes can be found in Table 3. There was a 2.1% divergence at cytochrome b across the whole sample set and a 0.475% divergence between haplotypes M and R representing the captive mhorr and ruficollis populations.

**Table 3:** Matrix of pair-wise mutational differences between cytochrome b haplotypes.

	1	2	3	4	5	6	7	8
**1**								
**2**	1							
**3**	2	1						
**4**	4	3	4					
**5**	3	2	3	1				
**6**	3	2	3	5	4			
**7**	3	2	3	5	4	2		
**8**	4	3	4	6	5	3	1	

## Discussion

The purpose of this study was to assess the relatedness and relative genetic diversity in extant populations of dama gazelle and to use this information to begin an assessment of the conservation value of different populations.

### Genetic diversity of dama gazelle populations

The mtDNA control region data show genetic diversity generally appears to be lower in captivity than in the wild populations (note that for samples exhibiting the same control region haplotype in the wild, we do not know to what extent resampling has occurred, but unintentional resampling would deflate not elevate diversity). Particularly striking is the much higher haplotype diversity in the wild populations as a whole (OROA_R, MANGA_R, TERMIT_D) compared with the captive and captive-derived *mhorr* populations (AIN_M, EEP_M, SEN_ M, SAF_M) and the captive *ruficollis* populations samples (AIN_R, MAR_R) (Figure 2). Low genetic diversity in captivity is unsurprising given what we know of the captive population's history (see Introduction). This survey represents a fairly representative sample of the captive *mhorr* population derived from the founding stock at La Hoya (represented by populations AIN_M, EEP_M, SEN_M & SAF_M). However, we have not been able to survey genetic diversity extensively within the captive *ruficollis* population, having only sampled from derived European (MAR_R) and Arabian (AIN_R) captive populations, and not the original population brought from Chad to the USA. We can expect USA populations to retain some additional genetic diversity not found in the derived populations sampled as part of this study. Understanding how the captive *ruficollis* population in the USA, both within zoos and private collections, relates to dama gazelle populations globally, represents an important future task. It is interesting to note that the animals sampled from the *ruficollis* captive population exhibit haplotypes that are closely related to those found at OROA (Figure 5), as indeed might be expected given the location that their ancestors were taken from (Figure 1), although see discussion of general lack of phylogeographic structure later. Genetic diversity in one of the wild populations (TERMIT_D) is equivalent to that of the captive *mhorr* populations. This may represent a worrying sign for this wild population, however mtDNA sequencing and the level of sampling available lack the resolution required to compare genetic diversity fully.

Captive populations of threatened species should be managed to maximise genetic diversity and to minimise inbreeding for two reasons: to reduce the likelihood of inbreeding depression, and to retain the greatest adaptive potential of the population.

Inbreeding has been shown on numerous occasions to have a detrimental effect on fitness in naturally outbreeding species [43]–[45] and review by [46]. This includes studies of captive populations [47]–[48] and populations released into the wild [49]–[51]. Pedigree estimates of inbreeding do not correlate with either body size or juvenile mortality in captive populations of *mhorr* at La Hoya ([52]–[53], although see earlier study [54] for contradictory results). It had been suggested that either captive conditions are artificially inflating fitness or that purging of deleterious alleles are responsible for this lack of effect. On the other hand, in the same population, genetic diversity, measured across 17 microsatellite loci (but not pedigree estimates of inbreeding), is correlated with semen quality, a trait that is directly related to fitness [55]. Therefore, Ruiz-Lopez *et al.* [55] suggest (based on results both from *mhorr* and a captive *Lynx pardinus*, population) that in highly inbred captive populations, pedigree estimates of inbreeding are often not reliable indicators of accumulated inbreeding, because base levels of inbreeding may already be high and assumptions of non-relatedness of founders may not be met. It seems likely that captive *mhorr* populations are suffering from other effects of inbreeding, but it is hard to measure these effects due to lack of statistical power. Similar studies have not been conducted for *ruficollis*.

Inbreeding depression is not the only reason to be concerned about inbreeding in captive dama gazelles. Concurrent loss of genetic diversity may result in loss of adaptive potential, limiting the ability of the population to evolve [56]–[58], to be resilient to environmental change or disease [59]–[60], and to retain the ability of readapting to wild environments from captivity [61]–[63].

Although genetic diversity in the wild appears to be considerably higher than in captivity, diversity is undoubtedly being lost in wild populations and this is of concern for the same reasons as mentioned above. Populations are small and isolated, and in the long term may become vulnerable to inbreeding, if the situation remains the same. Interestingly no haplotypes were shared between different populations despite lack of genetic structure (see below). This may indicate a lack of recent gene flow between sites and genetic drift in small fragmented populations, or may simply be an artefact of the small sample sizes available to this study. Additionally, the comparatively small number of haplotypes found in Termit versus the other wild populations (Figure 3) may indicate elevated levels of inbreeding there. For example twice as many samples were collected from Termit as from Manga but half as many haplotypes were found. The small sample sizes involved make it hard to draw any strong conclusions (Figure 3). A critical task for dama gazelle conservation is to secure genetic diversity and population connectivity in the wild. However, hunting, habitat loss and competition for grazing due to pastoral development and inherent vulnerability to demographic fluctuations caused by small population size are likely to be more immediate threats to the species' survival than loss of genetic diversity [12].

### Relatedness of dama gazelle populations

A crucial task for this study was to make an assessment of the relatedness of dama gazelle populations. Owing to its desperate conservation status, opportunities for sampling in the wild are limited. Despite this issue, a pattern emerged that suggests that phylogeographical structure of mtDNA across the range of the dama gazelle is weak or even absent altogether (Figure 5). There is a low level of support for basal nodes of the control region and cytochrome b trees (Figure 4), which means that it is not possible to resolve the entire structure of interrelatedness of the mitochondrial genome with confidence, but there is higher confidence in substructures within the trees, which cluster together haplotypes from disparate geographical locations. For example, control region haplotypes A and B, which come from OROA and Manga, are supported with 98% bootstrap (NJ) and 94% posterior probability (Mr Bayes), and differ by only 3 bp mutations. Haplotypes E and F, which come from the captive *mhorr* population (originating from Western Sahara, see introduction) and Manga, are supported by 89% bootstrap/75% posterior probability and differ by 8 bp mutation steps. Haplotypes G and M from Manga, and the captive *mhorr* population, cluster with 83% posterior probability (no NJ support) and differ by 12 base pair mutations (Figure 4 & 5, Table 2).

Critically, the clustering of these groups is also supported by the pattern of relatedness at the more slowly evolving cytochrome b gene, because closely related control region haplotypes are associated with identical or closely related cytochrome b haplotypes (Figure 4, 5). Notably the closely related control region haplotypes M (*mhorr*), F (*mhorr*) and G (*ruficollis*/Manga) are associated with an identical cytochrome b haplotype.

The result of examination of both control region and cytochrome b data is that the putative *ruficollis* subspecies grouping is likely to be polyphyletic with respect to both *dama* and *mhorr* (Figure 4), although support for the arrangement is not conclusive (75% posterior probability/89% bootstrap). Sequencing of a larger section of the mtDNA or addition of more samples to the tree might result in greater resolution in the future.

Lack of phylogeographical structure at mtDNA genes has been found in two other large Saharan mammals across their range: the Dorcas gazelle, *Gazella dorcas* [8], [64] and the African wolf *Canis lupus lupaster* [65]. Other genetic studies of large mammals native to the Sahara are absent, perhaps because there are few or no animals left to study [14]. Evidence for phylogeographical structuring does exist in smaller non-flying vertebrates native to this region and is particularly associated with mountains ranges and micro-scale water features [66].

### Phenotypic variation

The lack of phylogeographical structure across the dama gazelle range does present a contradiction because the cline in pelage coloration (Figure 2) points to the fact that some level of genetic divergence must be present, assuming that coat-colour is a trait under genetic control. The existence of clines in phenotypic traits is widespread and well documented (for review see [67]–[68]). Clines in coat coloration in the oldfield mouse (*Peromyscus polionotus*) and rock pocket mouse (*Chaetodipus intermedius*) have been linked to variation in substrate colour exerting differential selection pressure on specific genes [69]–[70] and micro-habitat variations in enivormantal luminosity have been linked to divergence in the sympatiric mtDNA clades of the Saharan jerboa (*Jaculau jaculus*) [71]. The case of the oldfield mouse has interesting parallels to that of the dama gazelle: The mouse inhabits a sandy habitat and the coat colour polymorphism in question is a gradation in the extent of a dark cape over a pale body, with similarities to that of dama gazelle. This trait has been linked to selection on the regulatory region of the *Agouti* gene, a gene that has also been implicated in coat colour variation in a large number of mammals, including Soay sheep, *Ovis aries* [72], dogs, *Canis familiaris*, [73] and other domesticated animals [74].

There are distinct climatic differences between the Sahel and the Atlantic Sahara [66], which may be responsible for variation in coat colour as the darker coat coloration is roughly concordant with the Atlantic Sahara [15]. However it is not known how closely correlated trends in climate are to trends in phenotype (and note for example the relatively minor change in extent of coloration between animals originating from Western Sahara and those from Termit in the Sahel approx. 2800 km inland from the coast, Figure 2). It is also not known whether dama gazelles display other phenotypic traits (e.g. skeletal measures, behavioural traits) that are coincident with the change in pelage colour.

If the phenotype is not under selection, one possible explanation for the observed variation in pelage coloration is that genetic drift occurred in separate refugia, perhaps either side of palaeolake Mega-Chad, which was larger than the Caspian Sea 5,000–6,000 years ago and has undergone changes in size associated with climatic cycles for at least the last 23,000 years [75]. It is possible that divergence resulted in evolution of separate phenotypes, which came together again after a period of isolation, forming a broad zone of secondary contact. The apparent absence of structure at mtDNA loci does not support this theory. However, there may be a cline at nuclear markers, but not at mtDNA markers. Species with male-biased dispersal (which is often the case in mammals, although nature of dispersal is unknown in the case of the dama gazelle) have paradoxically higher rates of mtDNA intogression than at nuclear loci [76], and so it is possible that differentiation at mtDNA markers might disappear first and that we are viewing the results of extensive introgression over a very large area.

It seems unlikely however that pelage coloration is neutral to selection given the (historical) presence of predators [14] and perhaps more importantly, the high level of solar radiation present in the Sahara/Sahel region. The desert-dwelling antelopes, addax (*Addax nasomaculatus*) and Arabian oryx (*Oryx leucoryx*), are thought to have evolved a predominantly white pelage to reflect solar radiation for thermoregulation and advertisement when in the open and to aid with camouflage when in the shade [77]–[78]. Does the varying degree in white present on the dama pelage point to a varying need to reflect solar energy? Or perhaps a tension between this and camouflage either as adults or juveniles? Whether secondary or primary in origin, it is likely, given the mtDNA results that the cline in pelage is caused by selection on a small number of genes. Importantly considerable variation is apparent in pelage within populations (localities) [20] and to our best knowledge transition in phenotype gradually occurs across a very large area [15], suggesting a concurrent gradual change in selection pressure if one exists.

Until evidence to the contrary is produced, phenotype should be assumed to have some selective relevance as this is the most conservative scenario for conservation. The key here is that it is the preservation of the diversity of genes controlling phenotype that is important as this is the raw material on which selection can act (in different environments), not the phenotype *per se*. Genetic management of dama gazelles should not seek to deliberately breed for phenotype as this will certainly result in further inbreeding of captive populations and may lead to selection for unforeseen traits, because genes controlling coat colour are known to have pleiotropic effects on other (often fitness-negative) traits [74].

### Conservation Dilemma

The issues behind the apparent cline in dama gazelle phenotype come to the fore when dealing with the conservation of its populations, creating a classic dilemma for modern-day conservation: are the now fragmented (wild and captive) populations distinct enough to justify being managed separately?

By mixing populations that are distinct, the risk is that the population will suffer from hybrid sterility or outbreeding depression. The risk of maintaining them separately is inbreeding depression and loss of genetic diversity (see above). Frankham *et al.* [4] argued that when considering the option of mixing different populations for conservation purposes, the risk of outbreeding is generally much lower than the risk of inbreeding (see above), but that conservation practitioners tend to overplay the former risk. Predicting the probability of outbreeding depression in advance is not an easy task and Frankham *et al.* [4] propose the use of a flow chart for evaluating risk, where the risk of outbreeding depression is smaller with the absence of chromosomal differences, absence of gene flow for <500 years, and lack of substantial environmental differences between the populations (Figure 1 of [4]).

In the case of the dama gazelle it seems likely the range has been contiguous within the last 500 years and dama gazelles are known to migrate seasonally in search of water [26], [79] suggesting high connectivity (also suggested by mtDNA data). As environmental differences have not been determined, it is tempting to suggest that there is little variation, but given that phenotypic variation is present, we cannot be sure. There is some evidence of karyotypic differences between male animals from Almeria (*mhorr*) and San Diego (*ruficollis*) in the form of a single chromosomal rearrangement (Robertsonian translocation, centric fusion) between the short acrocentric Y_1_ male-specific sex chromosome and an autosome of pair 14 in the San Diego animals. However a karyotypic polymorphism is also present within animals from Almeria (*mhorr*); in this case a Robertsonian polymorphism of chromosome 1 [80]–[81]. A complex of four Robertsonian translocations was also found in a female *mhorr* in a private collection at Taif in Saudi Arabia, although the origin of this animal is not known [82]. Chromosomal translocations are commonly responsible for reproductive isolation, but polymorphism is known to be common in gazelles and has been observed in *Gazella subgutturosa*, *G. gazella* (from northern Israel), *Eudorcas thomsonii* and *Antilope cervicapra* [80], [83]. In summary, karyotypic evidence is inconclusive, but the presence of karyotypic differences suggest that we must treat seriously the possibility of genetic incompatibility between the captive populations.

### The dangers of the sampling effect

It must also be noted that the captive animals studied here are descended from few individuals (see introduction) and thus represent a very narrow sampling of the former range. This “sampling effect” must always be borne in mind when comparing captive populations, not only for karyotype, but also for phenotype (which may have become more constrained owing to intentional or unintentional inbreeding) and genetic variation (at both nuclear and genetic markers), which will have been subject to extreme levels of drift. The only way to understand patterns of karyotypic, genetic and phenotypic variation is to examine them in the wild, a task which is all but impossible both for logistical reasons and due to widespread population extirpation and fragmentation. Even when examining nuclear structure in wild populations, care must be taken not to conflate recent drift events caused by population fragmentation with deeper historical substructure. The best examination would involve the use of large numbers of linked markers [84] in both current and historic (museum) samples, but this may not be feasible.

### Dama gazelle taxonomy and conservation

The problem with the historical preference for splitting into subspecies is that the burden of proof rests with the contemporary researcher. The null hypothesis becomes: “there are *n* subspecies” even if subspecies were not classified on a rigorous scientific basis. A large number of putative dama gazelle subspecies were recognised historically, based on rather limited evidence, which the paper by Cano [15] rationalised to only three based on the best available phenotypic and geographical data at the time. We would argue that in retrospect perhaps even this revision did not go far enough, owing to the apparent clinal and non-discrete nature of the variation. Today, although the genetic data do not prove polyphyly of the three subspecies conclusively (thereby disproving a null hypothesis of three putative subspecies), the joint evidence at the mtDNA control region and cytochrome b are suggestive of polyphyly and it is crucial to note that the data would not support a split into three subspecies from an *a priori* assumption of one. The levels of genetic divergence at cytochrome b (0.5% between captive *mhorr* and *ruficollis* haplotypes and 2.1% as a whole) are at within-species levels both for the best studied Saharan gazelle species, the dorcas gazelle [8], [64], and for other a mammalian taxa [85].

In the light of this study and the evidence presented here, we conclude with the following statements relevant to the conservation of the dama gazelle:

1. To the best available knowledge, phenotypic variation was originally more or less clinal across the species range. Today, we see phenotypic variation between populations, but also within captive and wild populations. Phenotypic differences between subspecies in captivity may be exaggerated by (in)breeding of the current populations from small numbers of founder individuals. Uneven and incomplete sampling of historical wild populations, and absence and fragmentation of contemporary populations, has in addition possibly presented a distorted view of the original phenotypic variation throughout the species' range. There is no *a priori* reason to divide the cline into three discrete units and lack of coincident mtDNA genetic structure and the possible polyphyly of the nominate *ruficollis* subspecies supports this view.
2. The conservation of the dama gazelle will be greatly advanced if it is considered a single species without subspecies division, even though it exhibits phenotypic variation. Under the “three subspecies view” artificial impermeable boundaries are erected and only individuals which conform to a particular phenotype from within the same subspecies should be bred together, and used for reintroduction and population augmentation. Under the “monotypic species view” there is a continuum of suitability of donors to a population, where all else being equal, the geographically most proximate population is the most suitable, but there is no *a priori* barrier to exchange between any populations provided the risks of exchange have been evaluated properly.
3. Inbreeding depression and loss of genetic diversity must be taken seriously during ongoing management efforts. Evidence for inbreeding depression in a captive population has already been found [55]. Captive populations of dama gazelle should be managed to maximise genetic diversity and minimise inbreeding. To achieve this aim, the continuation and improvement of the coordination and monitoring of captive breeding efforts across the world is vital.
4. Unless there is evidence to the contrary, phenotype should be assumed to be under some degree of selection in the wild as this is the most conservative scenario to conservation. However, no attempt should be made to breed or select for “a true phenotype” in captivity as this will result in further loss of genetic diversity and possibly unintended selection for traits linked to phenotype.
5. The possible risks associated with interbreeding of animals from phenotypically distinct and geographically distant populations should be taken seriously, however the potential conservation benefits of such interbreeding should be evaluated. Small-scale scientifically monitored experimental crosses of captive *mhorr* and *ruficollis* individuals should be conducted as part of a risk-benefit analysis for future actions [57]. This analysis will provide information to evaluate the possibility of more extensive mixing of captive populations and will contribute useful information if translocation of animals between wild populations or between the wild and captivity are to occur in the future.

## Supporting Information

Table S1 Details of the individual samples used in this study.
(XLSX)
